# Effect of cognitive-behavioral therapy on domestic violence and its consequences in transgender youth: a randomized clinical trial, parallel group study

**DOI:** 10.1186/s12888-021-03224-z

**Published:** 2021-04-26

**Authors:** Mahdieh Damanpak-Rizi, Farnaz Farnam, Parisa Khodakhah

**Affiliations:** 1 Department of Reproductive Health, Tehran University of Medical Sciences, Tehran, Iran; 2 Support center for Iranian transgender (MAHTAA), Tehran, Iran

**Keywords:** Transgender youths, Family violence, Cognitive-behavioral therapy, Mental health, Randomized controlled trial, Iran

## Abstract

**Background:**

Family violence against transgender people is a common issue and affects their mental health. Very few if any interventions have been designed to reduce family violence against transgender youths. This RCT will evaluate the effect of cognitive-behavioral therapy on the violent behaviors towards transgender people.

**Methods:**

This study is a randomized controlled trial conducted on 50 transgender youths with selected inclusion criteria in Iran. The intervention will be undertaken on the parents or guardians of these transgender youths in eight 1-h online sessions for the intervention group to increase their knowledge of gender dysphoria, to help control their anger regarding their offspring’s gender dysphoria and learn to manage stressful situations. The primary outcomes include frequency of family violence towards transgender youths and also parental conflict resolution tactics. Depression, anxiety, stress, self-esteem, suicidal thoughts, and suicide attempts in transgender youth are the secondary outcomes.

**Discussion:**

To the best of our knowledge, this is one of the first RCT on family violence against transgender youth in the world. Findings will help to provide better education and intervention for transgender parents to reduce violence against their children.

Results: N/A.

Conclusion: N/A.

**Trial registration:**

IRCT20120609009975N7 (08/03/2020).

## Background

Gender identity is defined as being male or female, which corresponds to one’s gender [1]. There is usually a harmony between sex and gender identity, but sometimes these two items are incompatible [2]. According to the Diagnostic and Statistical Manual of Mental Disorders, fifth edition (DSM-5), “the incongruity between a person’s experienced gender and the gender assigned to him or her at birth” leads to “Gender Dysphoria,” a term that replaced the previous “Gender Identity Disorder” in order to avoid stigma [1, 3]. The prevalence of diagnosis of trans women ranges from 5 to 14 in 100,000 and trans men from 2 to 3 per 100,000. Since not all adults seek hormonal and surgical treatment at specialized clinics, this rate is probably underestimated [3].

Transgender people face many problems include lack of social support, community rejection, physical and family violence. Among these problems, family violence is a common issue [1, 4–7]. Family violence defines as violence between family members, including violence between current or former intimate partners and acts of violence between a parent and a child, between siblings, or elder abuse [8]. Some families do not accept transgender youth because of the religious atmosphere and fear of disgrace and harassment of neighbors and other members of society, and they continuously treat their children with violence [9]. A report in 2010 showed that 60% of transgender people are subject to family violence [10]. According to a report in 2014 in Iran, only 20% of parents support their transgender youth; 10% do not care about this issue and choose to ignore it, and 70% of parents deal with anger when their child’s demand gender reassignment. These parents usually do not accept their child’s problems or respond with inhumanity, anger, and resentment [2]. In qualitative studies conducted by the research team (under submission), many of the transgender’s problems in society stem from family violence and lack of family support [11].

Several studies reported a high level of family violence against transgender youth, which can be lead to suicidal thoughts, drug abuse, running away from home, and high-risk behaviors; but very few if any interventions have conducted to reduce violence against these youths by working with potential perpetrators of violence [10, 12].

Some researches support CBT as an effective intervention for decreasing family violence against children [13–15]. CBT researches conducted on violent perpetrator parents showed decreased anger and anxiety levels, improved parenting, family interaction, and problem-solving behaviors. Despite the effectiveness of CBT in decreasing child abuse, more new studies need due to the outdated nature of existing studies [15]. Even when children do not participate directly in cognitive-behavioral therapy (sessions are for parents only), they will benefit from improving parents’ skills and reducing their violent behaviors [16]. Based on some studies’ results, parents’ physical punishment after cognitive-behavioral therapy has decreased; and children’s post-traumatic stress symptoms, depression, and behavioral problems reduced significantly [17]. This study is one of the first RCT on family violence against transgender youth globally and in Iran.

## Method/design

### Aim

#### Research hypothesis

1. CBT can reduce parents’ or guardian’s violence perpetration towards transgender youths

Primary Objective:

1. The main primary objective is the frequency of family violence towards transgender youth before and after the intervention.

2. Another primary objective is comparing the parents’ conflict resolution tactics about transgender youths before and after the intervention.

#### Secondary objectives

1. The secondary objectives are depression, anxiety, stress, self-esteem, suicidal thoughts, and suicide attempts changes in transgender youths after the intervention

### Study design

This trial is a parallel randomized, controlled trial with block randomization and a 1:1 allocation ratio. The project has been reviewed and approved by the Tehran University of Medical Sciences under the ethical approval code TUMS.FNM.REC.1398.201 on 2020/02/18 and funding grant number 98–3–100-45,678.

#### Study setting

The exact prevalence of gender dysphoria in Iran is not determined. Based on a survey that recorded the subjects referred to the Tehran Psychiatric Institute (TPI) from 2002 to 2009 with a diagnosis of gender dysphoria by two independent psychiatrists, the prevalence of Gender dysphoria in Iran considered 1:141000 (referral population considered people aged between 15 and 44) [18]. Iran is one of the first Islamic countries where gender reassignment has been legalized since 1964 and was second only to Thailand for the most gender reassignment surgeries in 2008 [19]. However, despite the legality of gender reassignment, transgender youth are still not sufficiently accepted in society due to cultural and religious pressures; and society, family, friends, school, and co-workers perpetrate violence against transgender people. The steps for obtaining allowance of gender reassignment surgery for transgender people in Iran are as follows: Request to Family Court; refer the person to the Legal Medicine Organization (LMO) by the court; evaluate hormonal and karyotype tests and at least 12 counseling sessions with an expert psychiatrist in LMO; approve gender dysphoria diagnosis by a psychiatrist; reapproved the diagnosis in LMO commission with attendance of an expert group, and refer the approved request in LMO commission to the court for the final decision. Only after court approval the transgender youth can change their identity card, have the desired sex clothes, and undergo gender reassignment surgery (although many transgender people have started hormone therapy many years sooner by a private physician or my own).

### Participants

**Fig. 1:**
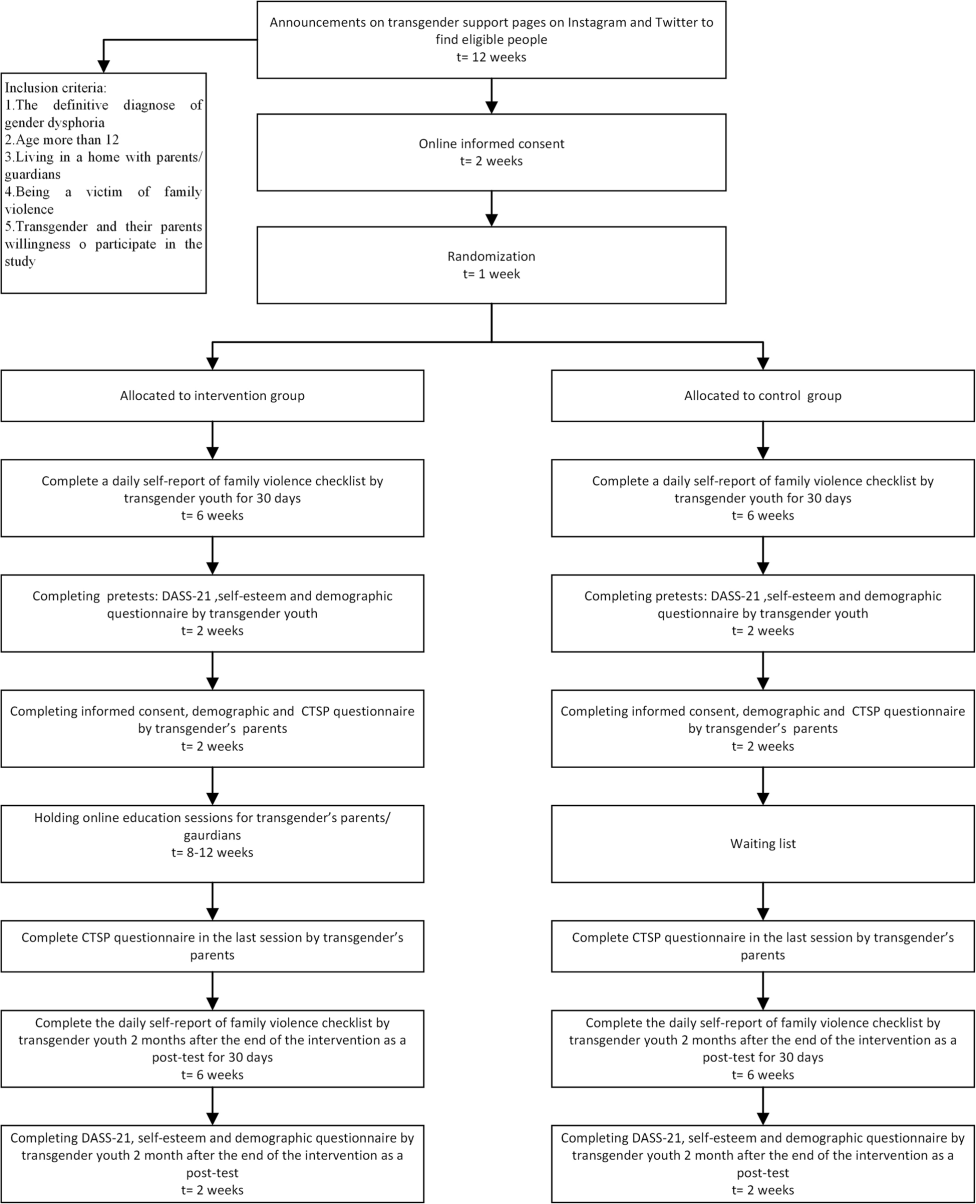
Participant timeline. Schedule of enrolment, interventions, assessments, and visits for participants

Our participants will randomly select from the list of transgender youth in the “support center for transgender Iranians “ (Mahtaa). An announcement about this study will first post on the Mahtaa pages on Instagram and Twitter. Inclusion criteria for transgender youths are the definitive diagnosis of Gender Dysphoria; age older than 12 years; living in a home with parents or guardians; transgender youth’s and their parents’ willingness to participate in the study and being a victim of family violence. This later criterion assesses through one question, “Have you experienced any physical, emotional, verbal, or sexual violence from your family in the past four months?” The only exclusion criterion is transgender parents’ unwillingness to attend classes during the intervention process. The eligible participant who intends to participate in the study will appraise the trial’s nature, and verbal and written informed consent will obtain. Our requisite sample size would be 50 transgender youth that will randomize into intervention and control groups using a randomized block design with four block sizes with a 1:1 allocation ratio. Study outcomes will measure through self-report questionnaires (Fig. 1).

#### Participant timeline

#### Randomization

The sample size of this study was calculated based on the most similar available article titled: “individual cognitive-behavioral treatment and family therapy for physically abused children and their offending parents: A comparison of clinical outcomes” [20]. Based on this article’s mean and standard deviation and with α = 0.05 and power 1-β 0.90 and considering a 20% drop, the total sample size required for each group is 25 people.

$$ N1=N2=\frac{\left(62+4.52\right)\left(1.96+1.28\right)}{\left(9-3.6\right)2}=21 $$

$$ {n}_1={n}_2=\frac{\left({S}_1^2+{S}_2^2\right){\left({Z}_{1-\frac{a}{2}}+{Z}_{1-\beta}\right)}^2}{{\left({X}_1-{X}_2\right)}^2} $$

FF will generate allocation sequence and opaque sealed envelopes of participants in each group for concealment and implementation mechanism. All people who give consent for participation and who fulfill the inclusion criteria will be randomized. Intervention sessions will be conducted by one of the research team members (MD) who is not involved in randomization and outcomes analysis.

Complete blinding is not possible due to the nature of the intervention. Neither participants nor staff can blind to allocation. An employee outside the research team will analyze data. Therefore, in this study, the outcome assessor will be blinded.

#### Intervention

Fifty eligible transgender youths will assign to the intervention and control group. For assessing the main primary objective (frequency of family violence), all transgender youths will complete the family violence self-report checklist daily for one month as a pre-test tool and again for one month as a post-test (2 months after ending intervention). DASS-21 and Rosenberg self-esteem questionnaires will be sent via an internet website for transgender youth as a pre-test and post-test for secondary outcomes. After completing the pre-tests by transgender youths, their parents will invite to the CBT intervention group. For assessing another primary outcome, the parents will complete Conflict Tactics Scales, Parent-child version (CTSPC) as the pre-test and at the end of intervention as post-test. After receiving pre-test questionnaires, our intervention (CBT) will begin with the parents of the transgender youths. Parents in the intervention group will attend eight 1-h online group therapy sessions. Furthermore, for better modification, these sessions will be held in four 2-h sessions if parents could not attend eight sessions. Two months after the last session of intervention, post-test questionnaires will send to transgender youths.

The first session of intervention contains information for increasing the parent’s/guardian’s awareness of the nature of gender dysphoria, with talking about the history of gender dysphoria in Iran and the world, the theoretical causes and risk factors, different reactions of parents, expressing the research team’s sympathy with the parents because of their sadness and concerns. The second session contains the problems faced by transgender youth, consequences of lack of family and community support, and examples of successful transgender youth. The third session includes an overview of existing counseling and treatments and health care needs before and after gender reassignment. The fourth session will be about the definition of violence, family violence, and anger symptoms. The fifth and sixth sessions contain anger management strategies, relaxation, and home exercises, and the seventh and eighth sessions include speech and discussion skills training, empathy skills, conflict resolution skills, and stressful conditions management.

For ethical consideration, the control group will receive all meeting content after ending intervention.

#### Outcomes measurement

##### Primary outcome measures

1. Family violence against transgender youth will be assessed by a research-made daily self-report checklist completed by transgender youths one month prior and two months after the intervention. In our research, family violence means the frequency of physical, psychological, verbal, and sexual violence perpetrated by a transgender parent or guardian [21].

2. Parental conflict resolution tactics will be evaluated by the “Conflict Tactics Scales, Parent-child version” (CTSPC). The basic form of CTSPC consists of 22 items divided into three scales: non-violent discipline, psychological aggression, and physical assault. The latter is further split into three subscales according to severity, which is respectively called corporal punishment, physical maltreatment, and severe physical maltreatment. The questionnaire also contains supplementary scales on disciplinary methods, neglect, and sexual abuse in the preceding week [22].

##### Secondary outcome measures

1. Depression, anxiety, and stress: The standard questionnaire of Depression Anxiety Stress Scale (DASS − 21) will use to determine these outcomes, It has 21 items, and each item has a 4-point Likert scale with a score ranging from 0 (“did not apply to me at all”) to 3 (“applied to me very much”) [23–25].

2. Self-esteem: Self-esteem will be determined by the standard questionnaire of Rosenberg Self-Esteem scale (RSES) [26], which has a score range between 0 and 30, where a score of less than 15 may indicate problematic low self-esteem. It is a ten-item Likert-type scale with items answered on a four-point scale—from strongly agree to disagree strongly. The scale measures global self-worth by measuring both positive and negative feelings about the self. (27)

3. Suicide thought and attempt: This outcome will be determined by two questions “How many times have you seriously considered suicide in the last four months? Never/once/ 2-3 times/4 times or more” and “How many times have you committed for suicide in the last four months? Never/once/ 2-3 times/4 times or more”.

##### Adherence

1. Researchers will call the transgender youth once a week to remind them to fill out the family violence self-report checklist in the pre-test and post-test period.

2. Researchers will call the transgender youth’s parents once a week to review any previous session issues and remind them of the next session.

3. Researchers will answer parents’ sub-questions about contraception, sexual disorders, and reproductive health at the end of the sessions to encourage them to continue meetings.

4. Researchers help participants to use internet websites for data collection.

##### Concomitant care

Parents or guardians who enroll in the study will be warned not to attend any family counseling from sampling and two months after the intervention.

#### Statistical methods

##### Outcomes

The intervention group will compare against the control group, and also, both groups will compare before and after the intervention. Data analysis will perform with SPSS 21. The first description of the data (in percentage and frequency, mean and standard deviation) will provide. And then by using Independent T-test (examining the questionnaire scores in terms of binary variables such as the study group and gender), paired t-test (Study before and after intervention in one group), Pearson correlation test (for correlating questionnaire scores, and quantitative variables such as age), ANOVA and Tukey post hoc tests (Analysis questionnaire scores and categorical variables such as job and economic status), Chi-square test (Relationship of two qualitative variables such as the relationship between levels of mental health and family violence with gender, marital status, ...) will analyze.

##### Additional analyses

For qualitative variables and quantitative variables with a cut-off point, logistic regression, and quantitative numerical variables, linear regression will use.

##### Analysis population and missing data

Outcome data obtained from all participants are included in the data analysis, regardless of protocol adherence. If there is missing data in questionnaires, they will return to participants for completion. Finally, if there is missing data, statistical methods of single imputation and multiple imputations will be used.

##### Access to data

MD and FF will give access to the cleaned data sets.

##### Data management

Electronic files of participants will be stored in numerical order and stored in a secure and accessible place and manner. Participant files will maintain in storage for two years after completion of the study.

##### Formal committee and interim analysis

This trial does not have a data monitoring committee and any stopping guidelines.

##### Harms

It seems that our RCT does not have any adverse effects on participants.

##### Auditing

The Tehran University of Medical Sciences will do auditing.

##### Protocol amendments

Any modifications to the protocol which may impact the conduct of the study, the potential benefit of the participants or may affect participants’ safety, including changes of study objectives, study design, participant population, sample sizes, study procedures, or significant administrative aspects, will be notified to the Ethics Committee of Tehran University of Medical Sciences.

##### Consent or assent

Comprehensible information about the research will provide in writing to the participants to ensure that they understand the research’s purpose and methodology and are fully satisfied with the study. Participants can discuss with the researcher if they have any questions to be left vague to them. Written informed consent will maintain until the end of the study.

##### Ancillary studies

Participants complete a consent form for using their data (without the name) in future research under FF supervision.

##### Confidentiality

All study-related information will be stored electronically. Each participant will have their code to fill out forms and questionnaires; therefore, all reports, data collection, process, and administrative forms will be identified by a coded number only to maintain participant confidentiality. All records that contain names or other personal identifiers will be stored separately from study records identified by code number. All files containing data will protect by password, and no one except the researcher will have access to it.

## Data Availability

The data will be available from the corresponding author on reasonable request.

## Abbreviations

CBT: Cognitive-behavioral therapy
TPI: Tehran Psychiatric Institute
LMO: Legal Medicine Organization
CTSPC: Conflict Tactics Scales; Parent-child version
DASS − 21: Depression Anxiety Stress Scale
RSES: Rosenberg Self-Esteem scale
